# Genetic mapping of high caries experience on human chromosome 13

**DOI:** 10.1186/1471-2350-14-116

**Published:** 2013-11-05

**Authors:** Erika C Küchler, Kathleen Deeley, Bao Ho, Samantha Linkowski, Chelsea Meyer, Jacqueline Noel, M Zahir Kouzbari, Mariana Bezamat, José M Granjeiro, Leonardo S Antunes, Livia Azeredo Antunes, Fernanda Volpe de Abreu, Marcelo C Costa, Patricia N Tannure, Figen Seymen, Mine Koruyucu, Asli Patir, Juan C Mereb, Fernando A Poletta, Eduardo E Castilla, Ieda M Orioli, Mary L Marazita, Alexandre R Vieira

**Affiliations:** 1 Department of Oral Biology, University of Pittsburgh, 614 Salk Hall, Pittsburgh, PA, USA; 2 Clinical Research Unit, Fluminense Federal University, Niterói, RJ, Brazil; 3 Directory of Programs, National Institute of Metrology, Quality and Technology (INMETRO), Duque de Caxias, RJ, Brazil; 4 Department of Specific Formation, School of Dentistry, Fluminense Federal University, Nova Friburgo, RJ, Brazil; 5 Department of Pediatric Dentistry and Orthodontics, Federal University of Rio de Janeiro, Rio de Janeiro, RJ, Brazil; 6 Veiga de Almeida University, Rio de Janeiro, RJ, Brazil; 7 Discipline of Cariology, School of Dentistry, Salgado de Oliveira University, Niterói, RJ, Brazil; 8 Department of Pedodontics, Istanbul University, Istanbul, Turkey; 9 Department of Pedodontics, Medipol Istanbul University, Istanbul, Turkey; 10 ECLAMC at Hospital de Area El Bolson, Rio Negro, Argentina; 11 ECLAMC (Latin American Collaborative Study of Congenital Malformations) at CEMIC (Center for Medical Education and Clinical Research), Buenos Aires, Argentina; 12 Department of Genetics, Oswaldo Cruz Foundation, ECLAMC at INAGEMP-CNPq (National Institute of Population Medical Genetics), Rio de Janeiro, Brazil; 13 ECLAMC at INAGEMP-CNPq (National Institute of Population Medical Genetics) in Department of Genetics, Institute of Biology, Center of Health Sciences, Federal University of Rio de Janeiro, Rio de Janeiro, Brazil; 14 Center for Craniofacial and Dental Genetics, and Clinical and Translational Science Institute, University of Pittsburgh, Pittsburgh, PA, USA; 15 Department of Pediatric Dentistry, School of Dental Medicine, University of Pittsburgh, Pittsburgh, PA, USA

**Keywords:** Caries, Genetics, Polymorphism, Oral health

## Abstract

**Background:**

Our previous genome-wide linkage scan mapped five loci for caries experience. The purpose of this study was to fine map one of these loci, the locus 13q31.1, in order to identify genetic contributors to caries.

**Methods:**

Seventy-two pedigrees from the Philippines were studied. Caries experience was recorded and DNA was extracted from blood samples obtained from all subjects. Sixty-one single nucleotide polymorphisms (SNPs) in 13q31.1 were genotyped. Association between caries experience and alleles was tested. We also studied 1,481 DNA samples obtained from saliva of subjects from the USA, 918 children from Brazil, and 275 children from Turkey, in order to follow up the results found in the Filipino families. We used the AliBaba2.1 software to determine if the nucleotide changes of the associated SNPs changed the prediction of the presence of transcription-binding site sequences and we also analyzed the gene expression of the genes selected based on binding predictions. Mutation analysis was also performed in 33 Filipino individuals of a segment of 13q31.1 that is highly conserved in mammals.

**Results:**

Statistically significant association with high caries experience was found for 11 markers in 13q31.1 in the Filipino families. Haplotype analysis also confirmed these results. In the populations used for follow-up purposes, associations were found between high caries experience and a subset of these markers. Regarding the prediction of the transcription-binding site, the base change of the SNP rs17074565 was found to change the predicted-binding of genes that could be involved in the pathogenesis of caries. When the sequence has the allele C of rs17074565, the potential transcription factors binding the sequence are *GR* and *GATA1*. When the subject carries the G allele of rs17074565, the potential transcription factor predicted to bind to the sequence is *GATA3*. The expression of *GR* in whole saliva was higher in individuals with low caries experience when compared to individuals with high caries experience (p = 0.046). No mutations were found in the highly conserved sequence.

**Conclusions:**

Genetic factors contributing to caries experience may exist in 13q31.1. The rs17074565 is located in an intergenic region and is predicted to disrupt the binding sites of two different transcription factors that might be involved with caries experience. *GR* expression in saliva may be a biomarker for caries risk and should be further explored.

## Background

Caries is a multifactorial disease and our ongoing research continues to provide evidence that genetic factors related to the host are involved in caries susceptibility. Our previous studies focused on genetic variation of genes involved in the enamel formation [1-4] and in the immunological system [5]. We complemented these studies by performing a genome-wide linkage scan to unravel novel loci for caries [6].

The genome-wide linkage study identified three loci for low caries experience (5q13.3, 14q11.2, and Xq27.1) and two loci for high caries experience (13q31.1 and 14q24.3) [6]. The fine mapping of the locus 5q12.1-13.3 suggested that *BTF3* has a functional role in the pathogenesis of caries [7]. The fine mapping of the locus 14q11.2 pointed at *TRAV4* as involved in caries experience [8]. Both genes are suggested to be protecting factors against caries. These results clearly demonstrate that focusing on the regions identified by the genome-wide linkage analysis can lead to the identification of genetic contributors to caries. In the present study we fine mapped the locus 13q31.1 in order to identify genetic contributors involved in high caries experience.

## Methods

### Studied population

We studied 3,151 individuals from six population data sets, including samples from the Philippines, USA, Brazil, and Turkey.

The Filipino sample set consisted of DNA samples from 477 subjects (224 females and 253 males) from 72 pedigrees recruited between 2005 and 2007 living in the Cebu Island. The mean age of the individuals was 25.8 years and ages ranged from one to 82 years. The mean DMFT/dmft score was 9.7 and scores ranged from 0 to 32. We compared individuals living in the same area in the Philippines, and therefore with similar cultural backgrounds and access to dental care, in an attempt to reduce the influence of environmental confounders. The families studied all come from the central part of the country, mostly Cebu Island, and the surrounding islands. All families are small-scale fishermen or landless rural dwellers. They all appear to be descendents from a proto-Malay stock. Most parents reported brushing the teeth of their children and similar dietary habits.

The sample from Pittsburgh, USA consisted of 1,481 (715 males and 766 females) unrelated subjects who sought treatment at the University of Pittsburgh and were part of the Dental Registry and DNA Repository project. The mean age of the individuals was 40.9 years and ages ranged from six to 92 years. The mean DMFT/dmft score was 15.9 and scores ranged from 0 to 28. This population is at high risk for oral and systemic diseases but no detailed data on caries risk factors are available for this study group. Pittsburgh is the largest city in the Appalachian region of the United States, which is one of the poorest in the country. Pittsburgh has had fluoridated water since 1953, however, nearly half of the children in Pittsburgh between six and eight have had cavities according to the State Department of Health of Pennsylvania (http://www.portal.state.pa.us/portal/server.pt/community/oral_health/14180). More than 70% of 15-year-olds in the city have had cavities, the highest percentage in the state. Close to 30% of the city’s children have untreated cavities. That is more than double the state average of 14%.

From Brazil, two sample data sets were available for this study. The first consisted of DNA samples from 598 unrelated children and teenagers (313 males and 285 females) that sought treatment at the Federal University of Rio de Janeiro during 2010 and 2011. The mean age of the children was 9.0 years and ages ranged from two to 18 years. The mean DMFT/dmft score was 2.5 and scores ranged from 0 to 17. The second sample set included DNA samples of children from Nova Friburgo recruited during the year of 2012. The city of Nova Friburgo is located in the northern mountainous region of the Rio de Janeiro state, 136 km from downtown Rio de Janeiro. Children (N =  320, 158 males and 162 females) were from eight daycare centers in Nova Friburgo. The mean age of the children was three and half years and ages ranged from one to six years. The mean dmft score was 1.4 and scores ranged from 0 to 16. Variables related to risk factors for caries were not available for all participants and these two Brazilian cohorts and these data could not be included in the analyses.

From Istanbul, Turkey, two sample data sets were also available for this study. The first sample was from a study originally designed as a case–control study and consisted of 172 unrelated children (93 females and 79 males) from three to six years of age recruited during the year of 2006. Ninety children had a dmft score of four or more and 82 children were caries free [2]. The second sample was designed as a cohort study and included 103 children (45 males and 58 females). The mean age of the children was five years and ages ranged from four to six years. The mean dmft score was 2.5 and scores ranged from 0 to 9. For this study group, most parents reported not brushing the teeth of their children. Drinking water in the region is not artificially fluoridated.

These samples were used with the approval of the University of Pittsburgh Institutional Review Board and each Institutional Review Board at the original sites where the samples were obtained (H.O.P.E. Foundation International Institutional Review Board, the Philippines; Federal University of Rio de Janeiro University Hospitals Research Ethics Committee, Rio de Janeiro, Brazil; Federal Fluminense University Research Ethics Committee, Nova Friburgo Brazil, and Istanbul University Institutional Review Board, Turkey) and appropriate informed consent was obtained from all participants. Age appropriate assent documents were used for children between seven and 14 years and informed; written consent was obtained from the child, as well as from the parents.

### Determination of caries experience

Caries was diagnosed using a modified World Health Organization protocol recommended for oral health surveys [9]. Teeth lost to trauma or primary teeth lost to exfoliation were not included in the final DMFT/dmft scores. When records indicated that teeth were extracted for orthodontic reasons or periodontal disease, or treatments were performed in sound teeth, these situations were not included in the final DMFT/dmft scores. The studies developed in Turkey included white spot lesions as evidence of caries. For all studies, carious lesions were recorded as present when a break in enamel was apparent on visual inspection. All the examiners carried out the clinical examination after being calibrated by an experienced specialist. Details about the determination of caries experience were previously described [1,2,4,6].

In this study, the populations were classified as either 'low caries experience’ or 'high caries experience’, based on DMFT/dmft distribution in each cohort (DMFT/dmft mean and standard deviation) and subject’s age. The criteria used here for classification of caries experience took age into consideration, since it is expected that caries experience will increase in the general population with age [10]. Table 1 presents caries experience definitions for Filipinos and US cohorts. For the Turkish and Brazilian cohorts (which included only children), subjects that had a DMFT/dmft score between 0–2 were classified as 'low caries experience.’ The subjects that had a DMFT/dmft score 3 or higher were classified as 'high caries experience.’

**Table 1:** Definitions of caries experience based on age and DMFT/dmft scores used in the Filipino, US, and Argentina data sets

**Philippines**			**USA**	**Argentina**
Caries Experience Level	DMFT/dmft	Caries Experience Level	DMFT/dmft	
Children [under to 12 years of age]		Children and teenagers [from 6 to 19 years of age]		
		Low caries experience	0-3	0
Low caries experience	0-2	High caries experience	4 or higher	1 or higher
High caries experience	3 or higher	Young Adults [from 20 to 39 years of age]		
Teenagers [from 13 to 19 years of age]		Low caries experience	0-10	0-2
		High caries experience	11 or higher	3 or higher
Low caries experience	0-5	Middle age [from 40 to 59 years of age]		
High caries experience	6 or higher	Low caries experience	0-15	0-5
Adults [20 years of age and older]		High caries experience	16 or higher	6 or higher
		Elderly [60 years of age and older]		
Low caries experience	0-8	Low caries experience	0-20	0-8
High caries experience	8 or higher	High caries experience	21 or higher	9 or higher

### Single Nucleotide Polymorphism (SNP) genotyping

A target region at the locus 13q31.1 was fine mapped based on our previous genome-wide linkage results [6]. This region covers approximately one million base pairs. For the selection of the SNPs we used data from the International HapMap Project on Whites and Chinese (http://www.hapmap.org), viewed through the software Haploview [11]. Based on pairwise linkage disequilibrium and haplotype blocks we selected 61 SNPs (Table 2) in the region and genotype was performed by polymerase chain-reactions with the Taqman method with the real-time PCR system ABI PRISM® 7900HT Sequence Detection System (Foster City, CA, USA). Probes were supplied by Applied Biosystems (Foster City, CA, USA).

**Table 2:** Single nucleotide polymorphisms (SNPs) and summary p-values of the association studies in the Philippines

**Marker ID**	**SNP**	**Minor allele frequency**	**p-value**	**Base pair position**
rs6563245	**CT**	0.271	**0.0000350**	chr13:82213394
rs4432145	**CT**	0.448	0.3542140	chr13:82225148
rs9545827	**AG**	0.194	0.0634870	chr13:82239682
rs17074565	**CG**	0.119	**0.0009550**	chr13:82242712
rs9531237	**AG**	0.375	0.4240030	chr13:82262814
rs9601669	**CT**	0.151	**0.0000140**	chr13:82267631
rs9545836	**AG**	0.189	0.0071440	chr13:82273953
rs2151504	**AC**	0.241	0.1266300	chr13:82291577
rs9318796	**CT**	0.298	0.0768220	chr13:82300870
rs9545841	**AG**	0.353	0.3168520	chr13:82309728
rs9531243	**AT**	0.461	0.4311590	chr13:82315374
rs7987364	**CT**	0.275	0.0997340	chr13:82320094
rs1490023	**CT**	0.012	**0.0008920**	chr13:82336749
rs9601697	**AG**	0.152	0.0218540	chr13:82350782
rs12429667	**CT**	0.206	0.0037260	chr13:82365765
rs9318803	**CT**	0.078	**0.0000045**	chr13:82390255
rs1565397	**CT**	0.008	0.0183477	chr13:82410045
rs9545880	**CT**	0.214	0.1434080	chr13:82413369
rs7987529	**CT**	0.472	0.3716050	chr13:82417111
rs9318814	**AG**	0.383	0.0059570	chr13:82451368
rs9545908	**AG**	0.375	0.7773880	chr13:82453046
rs9574982	**CT**	0.264	0.1193090	chr13:82467023
rs4112704	**AT**	0.140	0.0717210	chr13:82480825
rs7336983	**AG**	0.187	0.1013090	chr13:82485714
rs9601766	**CT**	0.128	0.0461800	chr13:82494257
rs9545915	**AC**	0.278	0.3908930	chr13:82498426
rs9593627	**GT**	0.331	0.0233040	chr13:82518251
rs17074923	**AC**	0.233	**0.0000619**	chr13:82518481
rs1280005	**CT**	0.026	0.0013520	chr13:82561205
rs1497062	**AT**	0.341	0.0831310	chr13:82581442
rs9531282	**AG**	0.001	0.0311380	chr13:82582150
rs2036130	**AG**	0.133	**0.0009210**	chr13:82606614
rs1331583	**CT**	0.313	0.0566020	chr13:82692849
rs9318840	**GT**	0.304	0.0566020	chr13:82770704
rs980635	**AC**	0.077	**0.0001120**	chr13:82869193
rs4497555	**AG**	0.400	0.0702320	chr13:82893029
rs9575086	**AG**	0.350	0.0035460	chr13:82913268
rs945359	**CT**	0.343	0.0020910	chr13:82946589
rs9546151	**AG**	0.004	0.3173110	chr13:83083854
rs9318882	**AC**	0.469	0.1216210	chr13:83127811
rs7322057	**AG**	0.263	**0.0000003**	chr13:83155775
rs1333586	**AC**	0.125	0.0122800	chr13:83183607
rs12869343	**GT**	0.294	0.0014180	chr13:83183712
rs12429646	**AG**	0.210	0.0191480	chr13:83207138
rs7990715	**AG**	0.473	0.0437580	chr13:83208055
rs9531374	**AG**	0.397	0.6243800	chr13:83212401
rs12429573	**AG**	0.252	0.0192650	chr13:83222937
rs2840279	**AG**	0.368	0.3640430	chr13:83230001
rs7990920	**AG**	0.339	0.1372060	chr13:83243050
rs9601950	**CT**	0.483	0.4572350	chr13:83268220
rs9546224	**AT**	0.314	0.1523830	chr13:83285405
rs12874183	**AT**	0.014	0.0253470	chr13:83293568
rs9593705	**CT**	0.170	0.0041870	chr13:83294037
rs7319120	**CT**	0.400	0.1160700	chr13:83314434
rs7999400	**AG**	0.311	0.0441200	chr13:83322249
rs9601986	**AT**	0.201	**0.0000260**	chr13:83401098
rs4885849	**CT**	0.085	**0.0000190**	chr13:83414814
rs9565817	**AG**	0.304	0.1260220	chr13:83423770
rs7983443	**CT**	0.436	0.6538970	chr13:83430208
rs982568	**CT**	0.429	0.4828760	chr13:83438876
rs11617124	**AC**	0.251	0.0877580	chr13:83443386
rs1906326	**AG**	0.491	0.0210680	chr13:83451878

Note: Bolded number indicates a statistically significant value.

For the first step of the genotype analyses, we evaluated the 61 selected SNPs in the Filipino families. The association between caries experience and the SNPs were tested with the transmission disequilibrium test (TDT) within the programs Family-Based Association Test (FBAT) under a recessive model [12], since the original linkage results [6] suggested a recessive model. After Bonferroni correction (0.05/61), an established alpha was 0.00082, to accommodate for the concern of multiple tests. In the second step of the genotyping analyses, we follow-up the results of eleven SNPs selected from the original 61 SNP panel based on obtained p-values. The data sets from the US, Brazil, and Turkey were tested. The differences in genotype and allele frequencies between 'high’ and 'low’ caries experience groups were tested using PLINK with an established alpha of 0.05. Haplotype analysis was also performed. Hardy-Weinberg equilibrium was evaluated using the chi-square test within each SNP in each population and only the results that were in Hardy-Weinberg equilibrium were further analyzed.

### Bioinformatics analysis to predict transcription factor binding sites

Since the 13q.31.1 region studied contains no genes, sequences containing the eleven associated SNPs were analyzed with AliBaba 2.1 software (http://www.generegulation.com/pub/programs/alibaba2/index.html).

This analysis was performed for the identification of an alteration of the prediction of potential transcription factor binding sites according to the base change of each SNP.

### Gene expression analyses

DNA and RNA extracted from whole saliva were used to assess expression levels of genes selected from the bioinformatics analysis. These samples came from 143 unrelated individuals living in twelve sites of the Patagonian region of Argentina recruited for two weeks, one during the month of December 2006 and the other during the month of May 2008, and are detailed elsewhere [7,8]. Samples are part of the University of Pittsburgh Center for Craniofacial and Dental Genetics studies. The mean age of the subjects was 21.7 years (between 1 and 72 years) and both the Centro de Educación Médica e Investigaciones Clínicas “Norberto Quirno” (CEMIC) and University of Pittsburgh Institutional Review Boards approved the study of these samples and appropriate written informed consent was obtained from all participants (parents provided consent for the participation of individuals 17 years of age and under). The criteria used for classification of caries experience are presented in Table 1.

Quantitative real-time PCR was used to determine expression in whole saliva of *GATA1*, *GR*, *GATA3*, *IL4*, *IL5*, and *IL13* genes (Table 3). *GATA1*, *GR*, and *GATA3* were selected because they are predicted to bind in the sequence affected by the SNP rs17074565. *IL4*, *IL5*, and *IL13* were selected due to evidence that *GATA3* can promote secretion of these genes [13].

**Table 3:** Primers used for mutation search and gene expression analyses

**Primers used for mutation search**				
**Primer**	**Primer sequences (5′–3′)**		**Amplicon size (base pairs)**	**Thermal cycling condition**
				
Forward	CAGCTTTATCGCCAGAGTCC		350	94°C 5 min→ 35×[94°C 30 sec, 56°C 30 sec, 72°C 30 sec]→72°C 5 min
Reverse	CCTCTTCCTCACCATCACCT			
Forward	AAGAAGGTGGGGAGGAAGAG		550	94°C 5 min→ 35×[94°C 30 sec, 53°C 30 sec, 72°C 30 sec]→72°C 5 min
Reverse	GGTCATCCGAGATCATTAAAAA			
Forward	TTTTTATACATATTGTTAGGGTCAGC		500	94°C 5 min→ 35×[94°C 30 sec, 53°C 30 sec, 72°C 30 sec]→72°C 5 min
Reverse	CAGTTTGTTTACCATCTCAAACACTT			
**Primers used for quantitative real-time polymerase chain reaction analysis**				
**Target Gene**	**Primer**	**Primer sequences (5′–3′)**	**Amplicon size**	**Thermal cycling condition**
			**(base pairs)**	
*GATA1*	Forward	TACTCAGTGCACCAACTGCC	114	50°C 2 min, 95°C 10 min→ 40×[95°C 15 sec, 60°C 1 min]→ 95°C 15 sec, 60°C 30 sec, 95°C 15 sec
	Reverse	CGGTTCACCTGGTGTAGCTT		
*GR*	Forward	AAGGGTTTGCTTTCACCCCA	138	
	Reverse	AAGCGTGTTGCAATTTCCCC		
*GATA3*	Forward	GAGATGGCACGGGACACTAC	102	
	Reverse	CTGCAGACAGCCTTCGCTT		
*IL4*	Forward	TCTTCCTGCTAGCATGTGCC	113	
	Reverse	GGTGCACAGAGTCTTCTGCT		
*IL5*	Forward	AGCCAATGAGACTCTGAGGAT	116	
	Reverse	CAGTACCCCCTTGCACAGTT		
*IL13*	Forward	ATGCATCCGCTCCTCAATCC	78	
	Reverse	AGTGAGAGCATGACCGTGG		
*GAPDH*	Forward	ACCACAGTCCATGCCATCAC	452	
	Reverse	TCCACCACCCTGTTGCTGTA		

Parametric and nonparametric tests were used to compare differences in expression between high and low caries experience individuals. The Pearson or Spearman correlation tests were used to analyze the strength of the relationship between *GATA3* and interleukins (*IL4*, *IL5*, and *IL13*) to verify if there is evidence of co-expression of *GATA3* and interleukins in whole saliva.

### Mutation analysis

**Figure 1:**
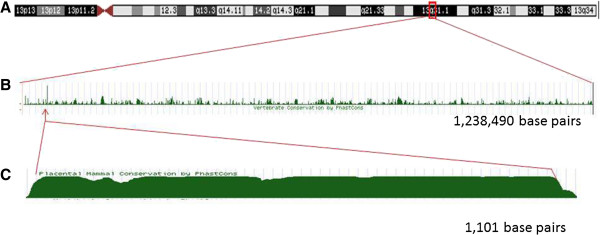
**Identification of a highly conserved sequence in 13q31.1.** Legend: **A**- Region in the chromosome 13 screened for a highly conserved sequence. **B**- The graph shows the level of nucleotide conservation across mammals, in which higher bars indicates higher conservation. **C**- Detail of the highest conserved region (data from the UCSC Genome Browser Feb. 2009 (GRCh37/hg19) assembly: http://genome.ucsc.edu).

We sequenced a region in 13q31.1 that is a highly conserved (Figure 1). This highly conserved area was identified by evaluation of data available at the UCSC genome browser (http://genome.ucsc.edu/). Three primers for the amplification of the entire region of approximately 1,200 base pairs were designed using the software PRIMER3. Primer sequences and PCR conditions are presented in Table 3. The sequences obtained were verified against a consensus sequence obtained from the UCSC genome browser with the software Sequencher 5.1.

## Results

### Association results in the Filipino Families

Out of 61 SNPs used for fine mapping the target chromosomal region, eleven were statistically significant or borderline associated (p ≤ 0.001) with caries experience. These results are summarized in the Table 4. Associations could also be seen between caries experience and the haplotypes of these markers (Table 5).

**Table 4:** Summary results of the associated markers according Family-Based Association Test (FBAT) in the Filipino families

**Marker ID**	SNP*	**MAF**^**#**^	**p-value**	**Position**
rs6563245	CT	0.271	0.0000350	chr13:82213394
rs17074565	CG	0.119	0.0009550	chr13:82242712
rs9601669	CT	0.151	0.0000140	chr13:82267631
rs1490023	CT	0.012	0.0008920	chr13:82336749
rs9318803	CT	0.078	0.0000045	chr13:82390255
rs17074923	AC	0.233	0.0000619	chr13:82518481
rs2036130	AG	0.133	0.0009210	chr13:82606614
rs980635	AC	0.077	0.0001120	chr13:82869193
rs7322057	AG	0.263	0.0000003	chr13:83155775
rs9601986	AT	0.201	0.0000260	chr13:83401098
rs4885849	CT	0.085	0.0000190	chr13:83414814

*Base change; ^#^Minor allele frequency.

**Table 5:** Summary results of the haplotype analyses in the 72 Filipino families

**Haplotype**	**n**	**Alleles**	**p-value**
rs636245-rs17074565	7	T-G	0.00505
rs17074565- rs9601669	10	G-C	0.00016
rs9601669-rs1490023	29	C-C	0.00001
rs1490023-rs9318803	8	C-C	0.00060
rs9318803-rs17074923	7	C-A	0.01201
rs17074923-rs2036130	16	A-A	0.00006
rs2036130-rs980635	17	A-C	0.00002
rs980635-rs7322057	14	G-C	<0.00001
rs9601986-rs4885849	24	A-C	<0.00001
rs17074565- rs9601669-rs1490023	18	C-C-C	0.00058
rs17074923-rs2036130-rs980635	12	A-A-C	0.00011

Notes: n = number of informative families.

### Association results in the follow-up Populations

Follow-up studies showed significant association for the markers rs17074565 and rs980635 in the Brazilian data set from Nova Friburgo and additional borderline results, which are presented in Table 6. A borderline result was found for the marker rs9601986 in a recessive model (p = 0.06) in the population data set from the US. In this same population, the genotype TT of the marker rs4885849 demonstrated to be a protect factor against caries in the logistic regression model (p = 0.029; OR = 0.26, 95% confidence interval 0.07-0.87). The logistic regression analysis included genotypes of the eleven markers selected after the analyses with the Filipinos as covariates.

**Table 6:** Summary results of the follow-up studies

**Markers**	**Pittsburgh (USA)**		**Rio de Janeiro (Brazil)**		**Nova Friburgo (Brazil)**		**Istanbul (Turkey)**		**Istanbul (Turkey)**	
	**Cohort**		**Cohort**		**Cohort**		**Case–control**		**Cohort**	
	**n = 1,481**		**n = 598**		**n = 320**		**n = 172**		**n = 103**	
	**Allele**	**Genotype**	**Allele**	**Genotype**	**Allele**	**Genotype**	**Allele**	**Genotype**	**Allele**	**Genotype**
rs6563245	0.49	0.58	-	-	**0.07**	0.21	0.30	0.17	0.75	0.73
rs17074565	0.32	0.56	0.98	0.98	0.68	0.05*	0.48	0.18	-	-
rs9601669	0.70	0.92	0.96	0.79	0.56	0.78	0.79	0.23	0.14	0.12
rs1490023	0.58	0.33	0.83	0.60	0.15	0.20	0.11	0.23	-	-
rs9318803	0.93	0.96	**0.06**	0.16	0.61	0.62	0.22	**0.06**	0.40	0.35
rs17074923	0.46	0.62	0.44	0.37	-	-	0.50	**0.07**	-	-
rs2036130	-	-	0.16	0.44	0.66	0.82	0.41	0.51	0.72	0.69
rs980635	-	-	0.80	0.91	0.33	0 .03*	0.85	0.65	0.72	0.75
rs7322057	0.94	0.95	0.20	0.08	-	-	-	-	0.15	0.25
rs9601986	0.13	0.16	0.66	0.82	0.98	0.82	0.90	0.55	0.47	0.73
rs4885849	0.26	0.29	0.31	0.59	0.55	0.17	0.39	0.33	0.93	0.62

Note: *indicates p ≤ 0.05; bold font indicates borderline results.

In the case–control study from Turkey, the marker rs9318803 was a protect factor for caries experience (p = 0.02; OR = 0.37, 95% confidence interval 0.16-0.85) in the logistic regression. An association with this same marker is the same population data set was also observed when the recessive model was tested (p = 0.03).

### Transcription factor binding site predictions according to the base change in each SNP

We determined potential transcription factors binding sites according to the base change of each associated SNP in the DNA sequence. Three of the eleven SNPs (rs17074565, rs9601669, and rs4885849) were predicted to alter the transcription factors binding to the sequence (Table 7). Prediction change for SNP rs17074565 were of particular interest. When the sequence has the C allele the predicted transcription factors binding are *GR* (glucocorticoid receptor) and *GATA1* (GATA binding protein 1). When the allele G is present, the predicted transcription factor binding is *GATA3* (GATA binding protein 3).

**Table 7:** Predicted transcription factor binding sites according to the base change

**rs number**		**rs17074565**	
**Flanking Sequence and base change**	cctggcagacaaa**C**agataatcattt	cctggcagacaaa**G**agataatcattt
	====NF-1==	====NF-1==
	=====GR===	===GATA-3=
	====GATA-1===	
**Potential transcription factors**		
**rs number**	**rs9601669**	
**Flanking Sequence and base change**	aggtgtaa**C**gagag	aggtgtaa**T**gagag
		===Oct-1==
**Potential transcription factors**		
**rs number**	**rs4885849**	
**Flanking Sequence and base change**	tctctttgact**C**tccttccata	tctctttgact**T**tccttccata
	====CPC1==	=REV-ErbA=
		===c-Rel==
**Potential transcription factors**		===LyF-1==

Note: Upper cases bold indicates base change.

### Gene expression in Whole Saliva

The genotype distribution of the SNP rs17074565 in the tested samples was 84 CC, 4 CG, and 10 GG. There was no association between caries experience and genotype distribution.

In the real-time PCR analysis, mRNA expression comparisons were performed between low and high caries experience groups, and according to genotypes. Statistically significant difference in the expression level of *GR* was found between low caries and high caries experience individuals (Table 8). No differences were found when genotypes and gene expression levels were compared between individuals with low and high caries experience (Table 9).

**Table 8:** Summary results of gene expression levels in whole saliva between individuals with low and high caries expression

**Gene**	**Tested groups**									
	***Comparisons between caries experience definitions***					***Comparisons between genotypes rs17074565***				
	**Low Caries**		**High Caries**			**CC**		**CG + GG**		
	**n**	**Mean ± standard deviation**	**n**	**Mean ± standard deviation**	**p-value**	**n**	**Mean ± standard deviation**	**n**	**Mean ± standard deviation**	**p-value**
*GATA1*	15	740.4 ± 653.7	20	515.3 ± 256.2	0.731	33	663.9 ± 346.8	5	22.6 ± 15.2	0.523
*GR*	41	41.1 ± 28.1	61	35.8 ± 9.1	**0.046**	80	76.8 ± 21.1	15	0.8 ± 4.6	0.448
*GATA3*	45	11.1 ± 37.5	70	27.1 ± 95.1	0.286	90	38.3 ± 150.1	17	3.4 ± 6.3	0.341
*IL4*	45	621.3 ± 3175.5	70	962.3 ± 5931.5	0.996	90	14.91 ± 75.9	17	1.9 ± 45.3	0.483
*IL5*	36	179.6 ± 779.3	52	2956.5 ± 16035	0.303	68	753.5 ± 641.3	11	25.1 ± 15.6	0.705
*IL13*	16	740.4 ± 2614.8	20	515.3 ± 1145.6	0.731	33	72.7 ± 220.1	5	0.9 ± 1.8	0.323

Note: bold form indicates p < 0.05.

**Table 9:** Comparison of gene expression levels depending on genotypes between individuals with low and high caries experience

**Gene expression**	**n**	**CC**	**n**	**CG + GG**	**P-value**
		**Mean ± standard deviation**		**Mean ± standard deviation**	
*GR*					
Low Caries	30	1.36 ± 6.2	6	0.16 ± 0.15	0.357
High caries	17	0.13 ± 0.72	7	0.90 ± 60.07	
*GATA1*					
Low Caries	14	839.53 ± 2793.54	2	1.68 ± 0.64	0.412
High caries	17	595.01 ± 1229.78	-	-	
*GATA3*					
Low Caries	33	13.48 ± 43.56	7	4.67 ± 8.07	0.601
High caries	50	33.17 ± 110.33	7	2.66 ± 5.82	
*IL4*					
Low Caries	33	12.17 ± 17.55	7	4.21 ± 10.48	0.910
High caries	50	18.07 ± 90.92	7	0.37 ± 0.58	
*IL5*					
Low Caries	27	207.1 ± 893.2	5	35.19 ± 75.47	0.839
High caries	36	1254.67 ± 7236.54	5	19.57 ± 25.98	
*IL13*					
Low Caries	14	84.29 ± 299.01	2	0.21 ± 0.09	0.886
High caries	17	70.04 ± 154.01	-	-	

*GATA3* expression was statistically significant correlated with *IL4* expression (r = 0.46; p < 0.0001), *IL5* expression (r = 0.23; p = 0.019), and *IL13* expression (r = 0.66; p < 0.0001).

### Mutation analysis

For mutation analyses, we selected 33 unrelated individuals from the Philippines that carried two copies of the associated alleles of markers rs6563245, rs17074565, rs9601669, rs1490023, and rs9318803. These markers were selected due to the proximity with the highly conserved region identified in the multispecies comparison (Figure 1). No mutations were found.

## Discussion

Our previous genome-wide linkage analysis showed suggestive linkage (LOD score above 2.0) to 13q31.1 when high caries experience was tested under a recessive model [6]. Our fine-mapping studies in the expanded data set of Filipino families confirmed the initial linkage results and showed association with markers in the locus. Eleven markers were over represented in allele transmissions to individuals with high caries experience. Follow-up studies of these eleven markers in five independent population data sets showed trends for association and associations between a subset of these markers in 13q31.1 and high caries experience. One possible explanation for the different results found in the Filipino samples in comparison to the other data sets is the possibility that the population from the Philippines studied here was more homogeneous, with very limited access to dental care, very similar diets based on rice and corn, and no exposure to fluorides and similar oral hygiene habits.

Since there are no genes in the studied region identified originally in our genome-wide linkage analysis, one of the intergenic SNPs in the region could in fact contribute to high caries experience. Another possibility is that the associated SNPs in the region could be in linkage disequilibrium to genetic variants outside the studied region, since the extent of linkage disequilibrium in the human genome can be greater than 100 kilobases [14-16].

One mechanism we are proposing is that the locus 13q31.1 may influence caries by altering transcription efficiency. The genetic variant (SNP) associated with high caries experience may disrupt a transcription factor-binding site. It is common knowledge that transcription-factors bind directly to DNA to cause changes in gene expression. To gain insight in this hypothesis we predicted transcription factors that bind to the sites of the SNPs associated with caries experience. Our analyses suggested that three markers (rs17074565, rs9601669, and rs4885849) potentially alter the prediction of the transcription factors binding to the region depending on the base change. Genes such as *OCT1* (organic caution transporters), *CPC1* (central pair complex 1), *c-Rel* (reticuloendotheliosis viral oncogene homolog), and *LyF-1* (IKAROS family zinc finger 1) were predicted to bind at the sequences of certain SNPs depending on the base change. The genes most likely to be involved in the pathogenesis of caries based on their function were related to the predictions made using the base change of SNP rs17074565. The C allele was predicted to have *GR* (glucocorticoid receptor) and *GATA1* (GATA binding protein 1) binding, but this prediction changed to *GATA3* (GATA binding protein 3) when the G allele was input. GR is the receptor to which glucocorticoids bind and there is evidence that show the use of anti-asthmatic medications with glucocorticoids decrease salivary flow rate and changes saliva composition and saliva pH [15-23]. In addition, rats receiving continuous glucocorticoid infusion show significantly increased caries progression, which may mean that glucocorticoids reduce the response of odontoblasts in the presence of a carious lesion. Our expression data show a statistically significant difference in *GR* expression when individuals with low and high caries experience were compared. The use of glucocorticoids to treat asthma is a likely mechanism that explains at least in part the evidence suggesting individuals with asthma have higher caries experience [24], however we have no record of our study samples came from individuals being treated with glucocorticoids. One possible reason we detected differential *GR* expression with higher levels in individuals with lower caries experience relates to individual salivary cortisol levels. Cortisol provides a quick burst of energy for survival purposes, heighten memory functions, burst immunity, lower sensitivity to pain, and helps maintain homeostasis. When secreted a higher levels for longer periods of time, it relates to a state of chronic stress. Salivary cortisol levels are found to be elevated in children with rampant caries and those levels will decrease after restorative treatment is provided [25]. Furthermore, children with early childhood caries have significantly higher levels of salivary cortisol when compared to caries free children, and a positive correlation of salivary cortisol levels of the mothers of children affected by early childhood caries exist [26], suggesting both child and mother salivary cortisol level may impact incidence of early childhood caries. Higher expression of *GR* in whole saliva of individuals with lower caries experience in our data may indicate these individuals have lower levels of stress, but also this may indicate a more active immune response system. Conversely, individuals with higher caries experience showing lower levels of *GR* expression in whole saliva may be more susceptible to cariogenic biofilm formation.

Our study has the obvious limitation of sample sizes that might not allow for the detection of relatively small effects. The frequency of the G allele of rs17074565 in the Argentina dataset is 12% and comparisons of relative gene expression stratified by genotypes were likely impaired in our data. However, our data also showed a correlation between *GATA3* levels of expression in whole saliva and expression of *IL4*, *IL5*, and *IL13* in the same individuals, which confirms previous findings [27]. Although the expression of these genes in whole saliva was not different depending on the caries experience in our data, it is possible that these genes may be involved in the pathogenesis of caries by the modulation of immune responses.

Another limitation is differences in caries definitions from the multiple study samples. The Turkish case–control cohort included white spot lesion as evidence of caries, while the other populations had carious lesions recorded as present when a break in enamel was apparent on visual inspection. Different definitions are due to the individual study designs that included examinations in a dental office versus in the house of the family participants, or the possibility of using compressed air to dry teeth. These differences can impact our results, particularly for the cohorts with lower caries levels (*i.e.*, from Brazil). One area of active research in our group is the determination of the best definition of caries for genetic analyses. The distribution of caries in the study population was different and we scaled low and high caries experience differently to accommodate those distinctions; hence, the different definitions in Table 1. The average caries experience level (mean DMFT scores) was used to help guide the determination of caries experience by age in each group.

## Conclusions

Genetic factors that contribute to high caries experience may exist in the gene desert 13q31.1 studied. rs17074565 may have a functional role in caries, disrupting the binding site for two different transcription factors that might be involved with immune responses. *GR* expression in saliva and salivary cortisol may be biomarkers for caries risk and should be further explored.

## Competing interests

The authors declare that they have no competing interests.

## Authors’ contributions

Manuscript writing: ECK (first draft of the manuscript), ARV (final draft of the manuscript). Data analysis: ARV, ECK. Study design: ARV, ECK, JMG, MCC, LA, FS, AP, IMO, EEC, MLM. Data collection: ARV, ECK, PNT, MK, AP, LA, LAA, FVA, JN, MZK, FAP, JCM. DNA/RNA manipulation/genotyping: ECK, KD, BH, SL, CM, LA, PNT, MB, AP. Critically reviewing the final draft of the paper: ECK, KD, BH, SL, CM, JN, MZK, MB, JMG, LA, LAA, FVA, MCC, PNT, FS, AP, MK, JCM, IMO, FAP, EEC. All authors read and approved the final manuscript.

## Pre-publication history

The pre-publication history for this paper can be accessed here:

http://www.biomedcentral.com/1471-2350/14/116/prepub
